# Molecular Epidemiology of Varicella‐Zoster Virus Among Patients From Two French Tertiary Care Hospitals: Differential Distribution of Clades According to Demographic Criteria

**DOI:** 10.1002/jmv.71198

**Published:** 2026-10-27

**Authors:** José Fernandez, Melinda Cheminet, Lucie Mazarguil, Mathilde Gay, Antoine Fauchois, Camille Tumiotto, Sonia Burrel, David Boutolleau

**Affiliations:** ^1^ Sorbonne Université, INSERM Sorbonne Public Health Institute Paris France; ^2^ AP‐HP, Sorbonne Université Pitié‐Salpêtrière University Hospital National Reference Center for Herpesviruses (Associated Laboratory), Virology Department Paris France; ^3^ Microbiology Department Créteil Intercommunal Hospital Créteil France; ^4^ Virology Department Université de Bordeaux, CNRS UMR 5234, Fundamental Microbiology and Pathogenicity Team, and Bordeaux University Hospital Bordeaux France; ^5^ Université Paris Cité, Faculty of Pharmacy Microbiology Department Paris France

**Keywords:** age, Clade 5, epidemiology, genotyping, Varicella‐zoster virus

## Abstract

Varicella‐zoster virus (VZV) nomenclature currently recognizes seven established clades (designated 1–6 and 9). A geographical segregation of VZV clades has been shown, with Clades 1, 3, and 6 predominating in Europe, Americas, and Australia, Clade 2 in Japan and East‐Asia, and Clades 4 and 5 in Asia and Africa. This distribution of VZV clades is dependent on the importation of viral strains, because of immigration and travels, and the implementation of varicella vaccine. The surveillance of VZV epidemiology is therefore necessary. VZV genotyping strategies rely on evaluating a panel of single nucleotide polymorphisms (SNPs) across the different open reading frames (ORFs) of the viral genome. We described retrospectively the VZV clade distribution among 339 patients in Pitié‐Salpêtrière (Paris) and Bordeaux University hospitals from 2018 to 2023. Clade 1 was detected in 27.1%, Clade 3 in 37.2%, Clade 5 in 31.9%, and other clades in 3.8% of the patients. The distribution of clades did not differ according to sex, immune status of the patients, and clinical presentation of VZV infection. However, patients infected with Clade 5 were statistically younger than those infected with other clades, especially European‐born patients. This epidemiological characteristic of VZV Clade 5 may have multifactorial etiology, including the combination of viral, host, and environmental factors.

## 1 Introduction

Varicella‐zoster virus (VZV) is a highly infectious human alphaherpesvirus. After primary infection, corresponding to varicella (chickenpox), the virus establishes lifelong latency in trigeminal and dorsal root ganglia. VZV latency is mainly controlled by VZV‐specific T cell‐mediated immunity, and when this immune response declines, because of aging or iatrogenic immunosuppression, VZV reactivation leads to herpes zoster (shingles). In rare cases, VZV infection can result in severe complications with ocular or central nervous system involvement [1, 2, 3]. The epidemiology of varicella differs according to climates and seasons. Thus, in temperate climates, varicella is a childhood disease, whereas it is more common in adults in tropical countries [3, 4]. Moreover, a seasonal variation of herpes zoster has been reported with a peak in summer [5]. Both varicella and herpes zoster can be prevented by vaccination. Varicella vaccine consists of the attenuated VZV Oka strain (belonging to Clade 2) commercialized under the names Varilrix and Varivax and is well tolerated [6]. Conversely to other countries, such as the United States or Japan, universal varicella vaccination of children is not recommended in France [7]. The recombinant zoster vaccine Shingrix, containing VZV glycoprotein E (gE), has been shown to reduce significantly the risk of herpes zoster in adults over 50 years [8].

To date, seven established clades (designated 1–6 and 9) and two provisional clades (VII and VIII) are recognized by VZV nomenclature. VZV genotyping strategies rely on evaluating a panel of single nucleotide polymorphisms (SNPs) across the different open reading frames (ORFs) of the viral genome [9, 10]. A geographical distribution of VZV established clades is observed: Clades 1, 3, and 6 are found mainly in Europe, North America, and Oceania, Clade 2 is dominant in Japan and surrounding countries, and Clades 4 and 5 are found in Asia and Africa [11, 12]. However, this worldwide distribution of VZV clades is dependent on different factors such as the importation of viral strains, with the increase of immigration and international travel, the implementation of varicella live attenuated vaccine, and the potential viral recombination events. For instance, studies have reported an increased circulation of viruses from Clade 5 [13]. The surveillance of VZV molecular epidemiology is therefore necessary.

The aim of this study was to investigate the distribution of VZV clades in clinical samples from patients experiencing varicella or herpes zoster in two different French tertiary care hospitals from 2018 to 2023, and to identify potential factors influencing this distribution.

## 2 Patients and Methods

### 2.1 Patients and Ethics

Patients attending Pitié‐Salpêtrière University Hospital (Paris, France) from January 2018 to December 2023 and Bordeaux University Hospital (Bordeaux, France) from January 2022 to December 2023 for whom VZV infection was diagnosed with by means of polymerase chain reaction (PCR) were identified from the laboratory information systems. Demographic and clinical data, including sex, age, birthplace, immunosuppression (transplantation, autoimmune disease, solid cancer, hemopathy, and HIV infection), type of VZV infection (varicella or herpes zoster), type of herpes zoster‐associated rash (dermatomal, multimetameric, and disseminated), neurological (meningitis or meningoencephalitis), and ocular (keratitis, uveitis, or retinitis) complications were collected retrospectively from medical charts of patients. This retrospective study was carried out in accordance with the French MR‐004 reference methodology of the Commission Nationale de l'Informatique et des Libertés (CNIL). The need for informed consent from patients was therefore not required. The study was recorded in the dedicated registry from Assistance Publique‐Hôpitaux de Paris (AP‐HP) under the number 20231213145523 and the name “GenoZ.”

### 2.2 Sample Processing and VZV Genotyping

Residual samples available after performing the molecular VZV diagnostic test (mainly vesicular swabs collected in viral transport medium) were stored at −80°C. Nucleic acid extraction from all samples was performed with the automated EMAG platform (BIOMERIEUX), according to the manufacturer's instructions. VZV genotyping was performed for clinical samples that displayed a positive result of VZV PCR with the real‐time HSV1&2 VZV R‐GENE kit (BIOMERIEUX) with a cycle threshold (Ct) value below 30 on the LightCycler 480 (Roche Diagnostics). VZV clade determination was based on the identification of seven targeted SNPs located in ORF21 (position 33725), ORF22 (positions 37902, 38055, 38081, and 38177), ORF38 (position 69424), and ORF50 (position 87841) in line with previous reports [10, 14, 15, 16]. Clade 2 VZV strains were further investigated in order to differentiate wild‐type from vaccine VZV strains by analyzing five additional vaccine‐specific SNPs located in ORF38 (position 69349) and ORF62 (positions 105705, 106262, 107252, and 108111) [17]. PCR amplifications were performed using the high‐fidelity enzyme PrimeSTAR GXL DNA Polymerase (TaKaRa), according to the manufacturer's instructions. Thereafter, amplification products were sequenced by Sanger method with the Prism Big Dye Terminator Cycle Sequencing Ready Reaction kit (Applied Biosystems) and analyzed with the automated sequencer ABI 3730 Genetic Analyzer (Applied Biosystems), using previously described procedures [18]. All nucleotide sequences were compared with that of VZV reference strain Dumas with GenBank accession no. X04370 [19] using SeqScape software v3.0 (Applied Biosystems), and identification of targeted SNPs allowed VZV genotyping.

### 2.3 Statistical Analysis

Continuous variables were expressed as median (interquartile range [IQR]) and categorical variables as numbers (percentages). Comparisons were performed using Mann–Whitney *U* test for continuous variables and Chi‐2 test for categorical variables. Multivariable logistic regression model was performed to evaluate confounding factors. Variables that had significance in the univariable analysis were added to the model. Association was expressed by odds ratio (OR) and 95% confidence interval (95% CI). *p* value below 0.05 was considered to be statistically significant. Statistical analyses were performed using GraphPad Prism (version 10; GraphPad Software, San Diego, CA) and StatView (version 5.0; SAS Institute Inc., Cary, NC).

## 3 Results

### 3.1 Baseline Characteristics of the Study Population

A total of 339 patients (226 from Pitié‐Salpêtrière University Hospital and 113 from Bordeaux University Hospital) were included in the study. Baseline characteristics of patients are presented in Table 1. A total of 179 (52.8%) males and 160 (47.2%) females, with a median age of 50 years [IQR, 29–69] were included. Patients originated mainly from Europe (*n* = 197; 58.1%) and Africa (*n* = 75; 22.1%). Immunosuppression was reported for 159 (46.9%) patients. Sixty‐six (19.5%) patients presented with varicella and 224 (66.1%) with herpes zoster. Patients with herpes zoster were statistically older than those with varicella (median age: 59.0 vs. 23.5 years; *p* < 0.0001) and more likely to be immunocompromised (63.4% vs. 15.9%; *p* < 0.0001). The frequency of patients with varicella was significantly higher in African‐born patients than in European‐born patients (30.9% vs. 13.4%; *p* < 0.0001), and African‐born patients with varicella were significantly older than European‐born patients with varicella (median age: 24.0 vs. 20.0 years; *p* = 0.024). Neurological and ocular complications were reported for 50 (14.7%) patients. Clinical samples for VZV genotyping consisted in mucocutaneous swabs (*n* = 288), peripheral bloods (*n* = 57), cerebrospinal fluids (CSFs; *n* = 16), bronchoalveolar lavages (BALs, *n* = 5), and ocular samples (*n* = 15). For 34 patients, several clinical samples (2–4) from different body compartments were collected and analyzed.

**Table 1:** Baseline characteristics of the study population.

Variables	Patients, *n* = 339
Age (years)	50 [29–69]
Sex	
Male	179 (52.8)
Female	160 (47.2)
Origin	
Europe	197 (58.1)
Africa	75 (22.1)
Asia	23 (6.8)
America	12 (3.6)
Not reported	32 (9.4)
Immunosuppression	
No	130 (38.3)
Yes	159 (46.9)
− SOT	52 (15.3)
− Autoimmune disease	39 (11.5)
− Solid cancer	28 (8.3)
− Hemopathy	23 (6.8)
− HIV infection	11 (3.2)
− HSCT	6 (1.8)
Not reported	50 (14.7)
Clinical presentation of VZV infection	
Varicella	66 (19.5)
Herpes zoster	224 (66.1)
− Dermatomal	112 (33.0)
− Multimetameric	16 (4.7)
− Disseminated	21 (6.2)
− Unknown	75 (22.1)
Not reported	49 (14.5)
Clinical complications of VZV infection	
None	289 (85.3)
Neurological^a^	21 (6.2)
Ocular^b^	27 (8.0)
Neurological and ocular	2 (0.6)
Clinical samples (*n* = 381)^c^	
Mucocutaneous swab	288 (75.6)
Peripheral blood	57 (15.0)
CSF	16 (4.2)
BAL	5 (1.3)
Ocular sample^d^	15 (3.9)

*Note:* Categorical variables are reported as numbers (%) and continuous variables as medians [interquartile range].

Abbreviations: BAL, bronchoalveolar lavage fluid; CNS, central nervous system; CSF, cerebrospinal fluid; HIV, human immunodeficiency; HSCT, hematopoietic stem cell transplantation; SOT, solid organ transplantation.

^a^ Meningitis or meningoencephalitis.

^b^ Keratitis, uveitis, or retinitis.

^c^ For 34 (10.0%) patients, several clinical samples (2–4) from different body compartments were collected and analyzed.

^d^ Corneal swabs (*n* = 12) and ocular humors (*n* = 3).

### 3.2 VZV Clade Distribution in the Study Population

The distribution of VZV clade among patients was as follows: Clade 1, 27.1%; Clade 2, 3.2%; Clade 3, 37.2%; Clade 4, 2.4%; Clade 5, 31.9%; and Clade 6, 2.7% (Figure 1). No significant variation of VZV clade distribution was observed over the 6‐year study period (data not shown). Among the 11 patients infected with Clade 2, 8 were Asian‐born and 2 were European‐born (the birthplace of the last patient was not specified). The 2 European‐born patients, aged 6 and 22 years, presented varicella, whereas the 11 Asian‐born patients had a median age of 71 years and presented herpes zoster. No VZV vaccine strains were identified among those patients. Regarding the 34 patients with multiple VZV‐positive clinical samples, identical VZV clades were identified in all samples from each patient.

**Figure 1:**
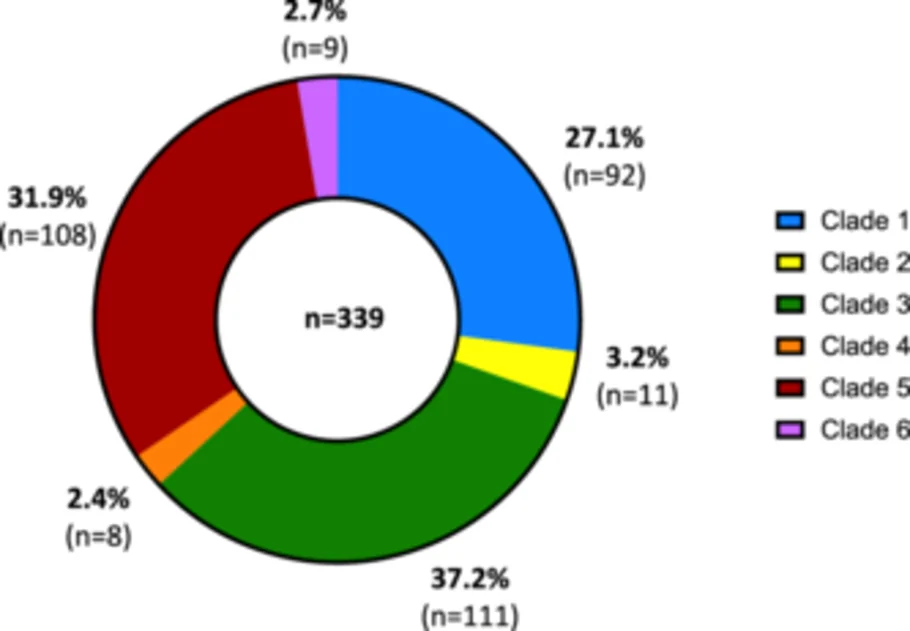
Distribution of VZV clades among patients.

### 3.3 VZV Clade Distribution in Relation to Clinical Characteristics of Patients

No significant association was found between VZV clades and clinical characteristics of patients. Thus, the distribution of clades did not differ according to the sex, the immune status of the patients, the type of VZV infection (varicella or herpes zoster), or the type of herpes zoster‐associated rash (dermatomal, multimetameric, disseminated). Clades 1, 3, and 5 accounted for the largest percentages of VZV genotypes identified in the 50 patients with neurological or ocular complications: Clade 1 (*n* = 13; 26%), Clade 3 (*n* = 20; 40%), Clade 5 (*n* = 13; 26%), and other clades (i.e., 2, 4, and 6) (*n* = 4; 8%). This clade distribution did not differ significantly from that observed among patients with no neurological nor ocular complications (Clade 1, 27%; Clade 3, 33%; Clade 5, 32%; and other clades, 8%). The same trend was observed when biological samples were considered. Thus, the clade distribution was similar between the 31 CSF and ocular samples and the 350 other types of samples (i.e., mucocutaneous swabs, bloods, and BALs) (data not shown).

### 3.4 VZV Clade Distribution in Relation to Demographic Characteristics of Patients

A significant association was found with the geographical origin of the patients: Clade 3 was significantly more frequent in European‐born patients (40.6%, *p* < 0.001), Clade 5 in African‐born patients (60.0%, *p* < 0.001), and Clade 2 in Asian‐born patients (33.3%, *p* < 0.001). Conversely, no significant geographical association was found for Clade 1 (Figure 2). A significant association was also found with the age of patients. Thus, as presented in Figure 3, patients infected with Clade 5 (median age, 41 years [24–62]) were statistically younger than those infected with Clade 3 (median age, 52 years [29–71]; *p* = 0.020), and Clade 1 (median age, 54 years [36–70]; *p* = 0.001) and other clades (median age, 59 years [43–72]; *p* = 0.008). Multivariable analysis confirmed that younger age (≤ 50 years) and African origin were independently associated with Clade 5 (OR: 2.21, 95% CI: 1.29–3.83, *p* = 0.004; and OR: 3.52, 95% CI: 1.34–9.32, *p* = 0.010, respectively).

**Figure 2:**
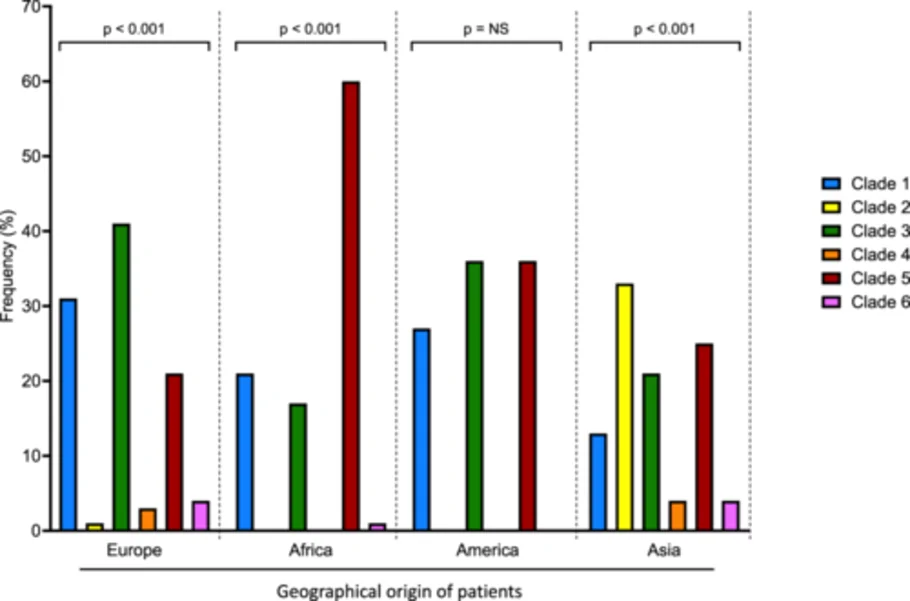
Distribution of VZV clades according to geographical origin of patients. Frequency (%) of VZV clades is represented according to geographical origins of patients. Comparisons were performed using Chi‐2 test.

**Figure 3:**
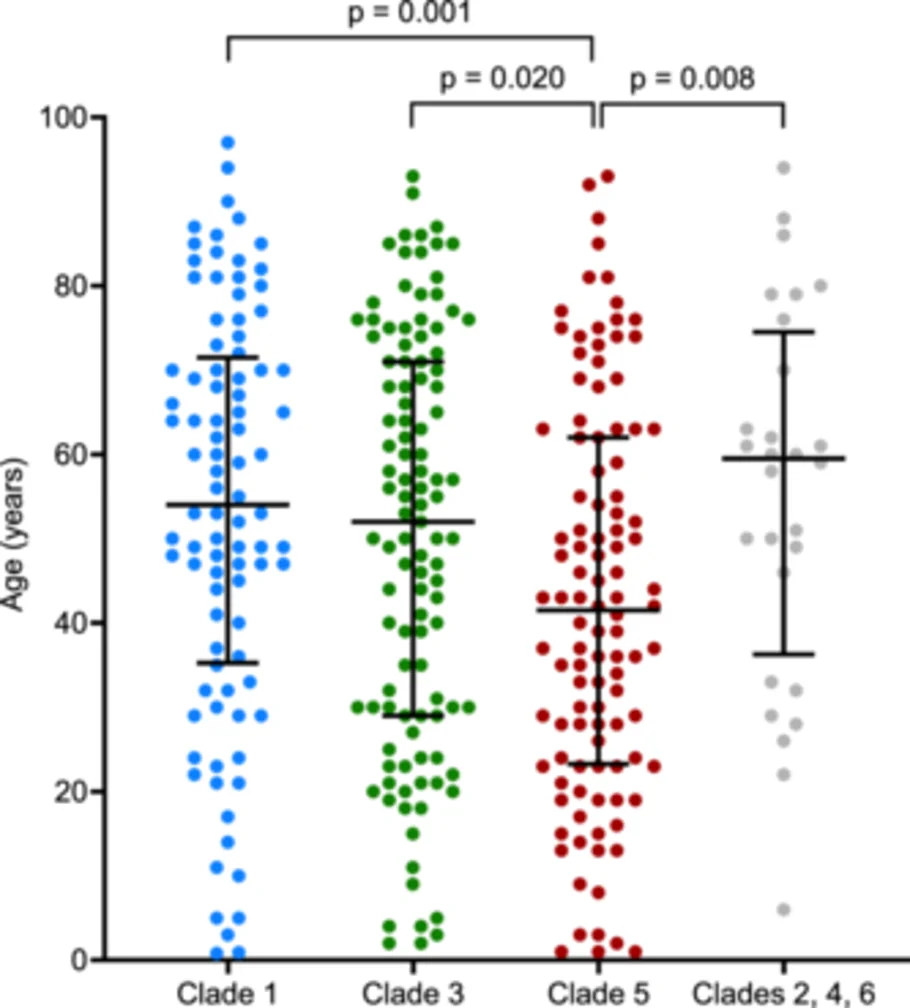
Comparison of patients' age according to VZV clades. Median (IQR) age (in years) is represented for patients infected with Clade 1, Clade 3, Clade 5, and Clades 2, 4, and 6. Comparisons were performed using the Mann–Whitney *U* test.

This difference of age remained when considering the type of VZV infection. Thus, among patients with varicella, those infected with Clade 5 (median age, 22 years [14–27]) were statistically younger than those infected with non Clade 5 (median age, 29 years [22–36]; *p* = 0.021). Similarly, among patients with herpes zoster, those infected with Clade 5 (median age, 50 years [37–69]) were statistically younger than those infected with non Clade 5 (median age, 62 years [47–76]; *p* = 0.018) (Figure 4). However, this differential age distribution of Clade 5 among patients with herpes zoster was dependent on the origin of patients. Among European‐born patients with herpes zoster, this difference remained. Patients infected with Clade 5 (median age, 48 years [27–62]) were statistically younger than those infected with non Clade 5 (median age, 63 years [48–76]; *p* = 0.005). Meanwhile among African‐born patients with herpes zoster, those infected with Clade 5 (median age, 55 years [44–73]) age were not significantly different than those infected with non Clade 5 (median age, 60 years [46–72], *p* = 0.93) (Table 2). Among patients with varicella, no significant age difference linked to the patient's origin between those infected with Clade 5 or non Clade 5 has been detected (data not shown).

**Figure 4:**
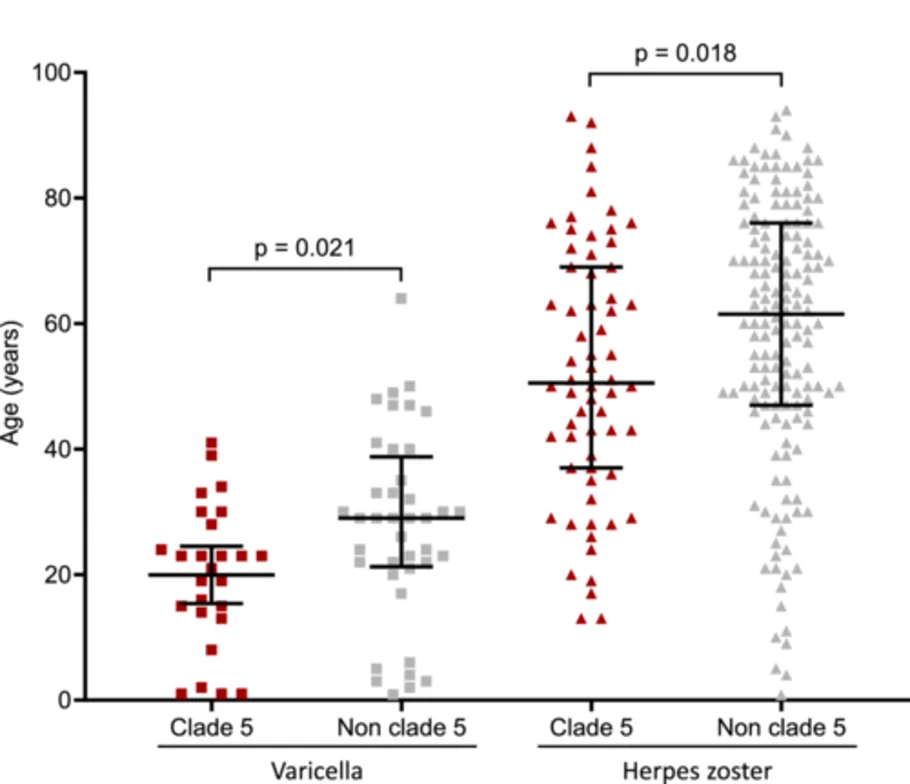
Comparison of patients' age according to VZV clades and type of VZV infection. Median (IQR) age (in years) is represented for patients experiencing either varicella or herpes zoster and infected either with Clade 5 or with non Clade 5. Comparisons were performed using the Mann–Whitney *U* test.

**Table 2:** Analysis of age of patients experiencing herpes zoster associated with VZV Clade 5 according to geographical origins.

	Median age [IQR] of patients		
	Clade 5	Non Clade 5	*p* value
All patients (*n* = 224)	50.5 [37–69]	61.5 [47–76]	**0.018**
Patients from Europe (*n* = 149)	48 [27–62]	63 [48–76]	**0.005**
Patients from Africa (*n* = 47)	55 [44–73]	60 [46–72]	0.93

*Note:* Significant *p* values < 0.05 are indicated in bold.

Abbreviation: IQR, interquartile range.

## 4 Discussion

In the present study, a molecular epidemiological study of VZV among 339 patients attending 2 different French tertiary care hospitals was performed, and the distribution of the different VZV clades could be analyzed according to various demographic and clinical factors of patients. The size of our study population was similar to the ones from previous VZV epidemiological studies conducted in Germany (*n* = 322) and in Spain (*n* = 294) [15, 20].

In our study population, as well established, patients with herpes zoster were older than those with varicella and more likely to be immunocompromised [1]. In addition, a notable disparity was observed. Compared to European‐born patients, African‐born patients with varicella were proportionally more significant (30.9% vs. 13.4%) and older (median age: 24.0 vs. 20.0 years). This difference may be the result of both climate and immigration consequences. Thus, climatic conditions are known to influence the transmission of VZV. Tropical and subtropical climates result in a lower transmission of VZV and therefore primary infections occur at older ages, as previously reported in studies from Africa, India and South East Asia and Africa [21, 22]. Therefore, VZV‐naive adult migrants coming from those countries to Europe are likely to meet the virus for the first time and get varicella.

The distribution of VZV clades in the study population was similar to the one from previous studies. Clades 1 and 3 were dominant in European‐born patients (31.0% and 40.6%, respectively) and Clade 5 was dominant in African‐born patients (60.0%) [11, 13, 23]. However, a significant frequency of Clade 5 was detected among European‐born patients (20.8%) as previously reported [20]. Eleven patients were infected with Clade 2, but no vaccinal VZV strain was identified. Previous studies reported some cases of infections with VZV vaccine strains in Germany (*n* = 3), Spain (*n* = 4), and the United States (*n* = 3) [14, 15, 20]. This finding may be explained by the public health strategy in France, where universal varicella vaccination of children is not recommended unlike those three countries [7]. In the present study, eight adult patients (median age, 62 years) with Clade 2 herpes zoster were born in Asia and were therefore probably infected at a young age by the predominant clade in their region of birth, and two young patients (6 and 22 years old) with Clade 2 varicella were born in Europe. No demographic data were available for the last patient infected with Clade 2. For the 34 patients with several samples (2–4) from multiple body sites, VZV clades obtained from distinct compartments were identical. These results are consistent with those obtained for 221 paired oral fluid/vesical fluid samples, and for sample combinations such as vesicle/plasma/BAL, vesicle/plasma/sputum, or vesicle/CSF [4, 24].

Among patients from the cohort study, the distribution of VZV clades did not differ according to sex, immune status, type of VZV infection (varicella or herpes zoster), and type of herpes zoster‐associated rash (dermatomal, multimetameric, and disseminated). Moreover, among VZV‐associated ocular or neurological complications, clade distribution was similar among the 31 CSF and ocular samples and the 350 other type of samples (mucocutaneous swabs, bloods, or BALs) in line with previous studies [14]. In our study, Clades 1 (26%), 3 (40%), and 5 (26%) were identified in patients with neurological or ocular complications in line with results obtained by Bryant et al. in CSF samples (Clade 1, 50%; Clade 3, 29%; and Clade 5, 13%) and Gonzalez et al. among patients with encephalitis (Clades 1 and 3, 56.4%) [14, 15].

Our study provided one insight of particular interest regarding Clade 5 epidemiological characteristics. VZV is a highly contagious virus that spreads rapidly within communities, especially during outbreaks in primary schools. With regard to the Clade 5, the transmission occurred likely occurred from children whose parents were from Africa to children whose parents were from Europe. It is even possible that some of those children have had siblings in childcare or preschool, places were varicella transmission in very likely, who were the source of contamination. This scenario is supported by the fact that the proportion of immigrant population is estimated to over to be over 10% in both Paris and Bordeaux. It is even possible that some of those children have had siblings in childcare or preschool, places were varicella transmission in very likely, who were the source of contamination. However, it is noteworthy that patients infected with Clade 5 were statistically younger than those infected with other clades, and this was observed both among patients with varicella and those with herpes zoster (Figures 3 and 4). This observation was previously reported in Germany for patients with varicella [20]. Moreover, we were able to show that this behavior of Clade 5 was dependent on patients' origin. Thus, among patients with herpes zoster, European‐born patients infected with Clade 5 were younger than those infected with non Clade 5 (median age, 48 vs. 63 years; *p* = 0.005), whereas this age difference was not observed among African‐born patients (median age, 55 vs. 60 years; *p* = 0.93). It is as if Clade 5, originating from the African continent [12], infected the population earlier and behaved differently among African and non‐African individuals. This difference could be attributed to specific viral or host characteristics. Indeed, few genetic characteristics of VZV have been reported to confer a specific viral phenotype, such as the single polymorphism D105N in the gE conferring an increased cell spread phenotype [25]. On the other hand, host characteristics may explain this phenomenon. For instance, mutations in human RNA polymerase III have been associated with VZV infection of the central nervous system [26], and human leukocyte antigen A (HLA‐A) molecules, with poor presentation capabilities of VZV immediate early 62 (IE62) protein, were more common in individuals with herpes zoster, especially those of young age [27]. At last, environmental factors have been reported to impact VZV transmission [3]. We could hypothesize that Clade 5, which evolved in tropical and subtropical regions, might compensate for its adaptation to temperate climate through more efficient transmissibility explaining the younger age of infected patients, and that it may reactivate under less restrictive conditions in European‐born patients, leading to herpes zoster earlier in life.

It is important to recognize the limitations of this study, which may not fully represent the epidemiology of VZV infections across France. Thus, in this retrospective study, the analysis is confined to cases referred to Pitié‐Salpêtrière and Bordeaux University Hospitals, potentially leading to an overrepresentation of immunocompromised individuals, particularly transplant recipients, and severe cases of VZV infections. Notably, herpes zoster infections were disproportionately prevalent compared to varicella, which may not be representative of the whole national population.

In conclusion, our results, together with those from Sauerbrei et al. [20], highlight the age‐related distribution of VZV Clade 5 among patients. This phenomenon is undoubtedly multifactorial in connection with different viral, host, and environmental characteristics. The molecular surveillance of VZV infections is needed to better understand the epidemiological evolution of VZV strains.

## Author Contributions

David Boutolleau and Sonia Burrel conceptualized the study. José Fernandez and Melinda Cheminet collected samples and conducted VZV genotyping experiments. José Fernandez, Melinda Cheminet, Lucie Mazarguil, Mathilde Gay, and Camille Tumiotto reviewed the medical charts and collected data. David Boutolleau, José Fernandez, and Antoine Fauchois analyzed data. David Boutolleau wrote the manuscript. All authors reviewed the manuscript and approved the final version.

## Conflicts of Interest

The authors declare no conflicts of interest.

## Data Availability

The data that support the findings of this study are available on request from the corresponding author. The data are not publicly available due to privacy or ethical restrictions.
